# Physiological and Biomechanical Responses to Cross-Country Skiing in Varying Terrain: Low- vs. High-Intensity

**DOI:** 10.3389/fphys.2021.741573

**Published:** 2021-10-11

**Authors:** Trine M. Seeberg, Jan Kocbach, Jørgen Danielsen, Dionne A. Noordhof, Knut Skovereng, Frédéric Meyer, Øyvind Sandbakk

**Affiliations:** ^1^Department of Neuromedicine and Movement Science, Centre for Elite Sports Research, Norwegian University of Science and Technology, Trondheim, Norway; ^2^Smart Sensor System, SINTEF DIGITAL, SINTEF AS, Oslo, Norway; ^3^Digital Signal Processing Group, Department of Informatics, University of Oslo, Oslo, Norway

**Keywords:** near-infrared spectroscopy, XC skiing, low-intensity training, inertial measurement unit, sub-technique detection, power, high-intensity training

## Abstract

The purposes of our study were to investigate the physiological and biomechanical responses to low-intensity (LI) and high-intensity (HI) roller ski skating on varying terrain and compare these responses between training intensities. Nine elite male skiers performed treadmill roller skiing consisting of two 21 min sessions (7 × 3 min laps) at LI and HI with the same set inclines and intensity-dependent speeds (LI/HI: distance: 5.8/7.5 km, average speed: 16.7/21.3 km/h). Physiological and biomechanical variables were measured continuously, and each movement cycle and sub-technique employed were detected and classified with a machine learning model. Both the LI and HI sessions induced large terrain-dependent fluctuations (relative to the maximal levels) in heart rate (HR, 17.7 vs. 12.2%-points), oxygen uptake ($\dot{V}O_{2}$, 33.0 vs. 31.7%-points), and muscle oxygen saturation in the triceps brachii (23.9 vs. 33.4%-points) and vastus lateralis (12.6 vs. 24.3%-points). A sub-technique dependency in relative power contribution from poles and skis exhibited a time-dependent shift from Lap 1 to Lap 7 toward gradually more ski power (6.6 vs. 7.8%-points, both *p* < 0.01). The terrain-dependent fluctuations did not differ between LI and HI for $\dot{V}O_{2}$ (*p* = 0.50), whereas HR fluctuated less (*p* < 0.01) and displayed a time-dependent increase from Lap 2 to Lap 7 (7.8%-points, *p* > 0.01) during HI. Oxygen saturation shifted 2.4% points more for legs than arms from LI to HI (*p* > 0.05) and regarding sub-technique, 14.7% points more G3 on behalf of G2 was employed on the steepest uphill during HI (*p* < 0.05). Within all sub-techniques, cycle length increased two to three times more than cycle rate from LI to HI in the same terrains, while the corresponding poling time decreased more than ski contact time (all *p* > 0.05). In sum, both LI and HI cross-country (XC) skiing on varying terrain induce large terrain-dependent physiological and biomechanical fluctuations, similar to the patterns found during XC skiing competitions. The primary differences between training intensities were the time-dependent increase in HR, reduced relative oxygen saturation in the legs compared to the arms, and greater use of G3 on steep uphill terrain during HI training, whereas sub-technique selection, cycle rate, and pole vs. ski power distribution were similar across intensities on flat and moderately uphill terrain.

## Introduction

Cross-country skiing is a demanding endurance sport that involves continuous changes in speed, external power, and energy system contributions while skiing across varying terrain. Added to the high aerobic metabolic power required to excel in cross-country (XC) skiing, sufficient anaerobic power and well-developed efficiency with associated technical and tactical skills are of high importance (Sandbakk and Holmberg, 2017). XC skiing is further complicated by continuous shifts between sub-techniques and the adaption of cycle rate (CR) and cycle length (CL) according to the topography of the track, during both training and competitions (Holmberg, 2015; Sandbakk and Holmberg, 2017).

For those reasons, training for XC skiers aims to improve their performance at high, competitive intensities, and even if the mean intensity is aerobic (i.e., 90–95% of $\dot{V}O_{2max}$), relatively substantial anaerobic contributions are shown to support aerobic energy delivery. There are large variations in the exercise intensity during races according to the change between uphill, flat, and downhill terrains. Several studies have shown work rates requiring ~110–160% of the maximal oxygen uptake ($\dot{V}O_{2max}$) of a skier on relatively short uphill segments during competitions (Gløersen et al., 2018; Karlsson et al., 2018; Losnegard, 2019), thereby combining nearly maximum aerobic energy delivery with significant amounts of anaerobic metabolic support. While such repeated bursts of high uphill work rates cause the substantial accumulation of fatigue, the strategy is possible due to the natural recovery allowed during subsequent downhill were external power requirements are nearly zero while flat segments also have smaller power requirements than in uphill (Losnegard, 2019).

Even though XC skiing competitions are performed at a high intensity (HI), with HI sessions regarded as a key stimulus in the development of XC skiers, most of the training is performed as long-duration sessions of skiing or roller skiing at low intensity (LI) (Sandbakk and Holmberg, 2017). LI training is regarded beneficial as it allows a large volume of training by keeping the degree of accumulated fatigue low (Sandbakk and Holmberg, 2017). However, LI sessions performed as XC skiing in varying terrain have been shown to induce significant terrain-dependent fluctuations in power output and heart rate (HR) (Bolger et al., 2015; Solli et al., 2018; Haugnes et al., 2019). As a result, the choice of sub-technique, kinematic patterns, and the loading of arms and legs will be challenged to various degrees depending on the terrain and intensity. Compared with most other endurance sports, those demands of XC ski-specific training and competitions are unique. Thus, this interval-based physiological and biomechanical stimulus in XC skiing, including the differences between LI and HI training, requires a more detailed elucidation.

In outdoor XC skiing, environmental conditions such as snow quality, air, and snow temperature, tracks, and wind influence skiing speed, choice of sub-technique, and cycle length, cycle rate, and the power distribution from arms and legs. This will, in turn, affect the metabolic demands. The combined use of various wearable sensors with adapted signal processing and smart classification and detection models stands to afford new, important insights into these interrelated physiological and biomechanical demands of XC skiing. For example, data from inertial measurement units (IMU) can be used to automatically detect XC sub-techniques and related macro and micro-kinematic patterns of skiers in the field without using resource-intensive methods such as video analysis (Seeberg et al., 2017, 2021; Rindal et al., 2018; Tjønnås et al., 2019). However, the few studies that have involved collecting such data to investigate LI vs. HI skiing were performed outdoors and limited by a lack of control over external conditions. To extend such work, a comprehensive understanding of the physiological and biomechanical demands of XC skiing under standardized conditions is necessary to subsequently take full advantage of those new possibilities in the field (Bolger et al., 2015; Solli et al., 2018; Haugnes et al., 2019). As an initial step, we, therefore, sought to complement an outdoor methodology with standard measurements available in the laboratory under standardized conditions using highly accurate reference sensors and methodology aiming to build competence on how to interpret data from sensors employed outdoors. Accordingly, the purposes of our study were to investigate physiological and biomechanical responses to LI and HI roller ski skating on varying terrain and to compare the responses between training intensities.

## Methods

### Overall Design

We measured physiological and biomechanical variables among elite skiers performing the same course at both LI and HI while roller ski skating on a treadmill. The data used represented a subset of a larger dataset of which parts have been presented in other studies with different aims and contexts (Noordhof et al., 2021; Seeberg et al., 2021). The protocol consisted of two consecutive parts performed on the same day with a 5 min recovery period in-between, each with the same preset load for all skiers: a 21 min LI bout to simulate a LI training session and a 21 min HI bout to simulate a mass-start or a HI training session. Initially, 13 elite male Norwegian skiers, consisting of eight XC skiers [distance International Ski Federation (FIS) points: 47 ± 21] and five biathletes, were recruited for the study and performed the protocol. The skiers who could not meet the required workload of the HI session were given one or more 30 s breaks (i.e., by grabbing a rope at the front of the treadmill, thereby simulating tuck) before they continued skiing the set protocol and only the nine skiers who completed the protocol without breaks were included in our study. $\dot{V}O_{2}$, HR, near-infrared spectrometry (NIRS), kinematics, and pole forces were monitored continuously, whereas blood lactate concentration (BLa) and rating of perceived exertion (RPE) were measured directly after each session.

### Participants

Anthropometric, physiological, and performance characteristics of the nine elite, male skiers included in the study are given in Table 1. All skiers, healthy and free of injury at the time of testing, were instructed to prepare in the same manner as before a competition, without performing strenuous exercise during the last 24 h before the test. The skiers were conversant with treadmill roller skiing and $\dot{V}O_{2}$ measurements from previous testing sessions and daily training routines.

**Table 1:** Anthropometric, physiological, and performance characteristics (*M* ± *SD*) of the nine male skiers in the study.

**Variables**	***M***	***SD***
Age [years]	25.9	2.2
Body height [cm]	185.7	6.7
Body mass [kg]	80.1	5.5
Body mass index [kg · m^−2^]	23.2	1.0
VO_2max_ [mL · min^−1^ · kg^−1^]	70.6	3.3
VO_2max_ [mL · min^−1^]	5,653	282
HR_max_ [beat · min^−1^]	191.7	7.3
Skinfold thickness of triceps brachii (arm) [mm]	6.1	1.6
Skinfold thickness of vastus lateralis (leg) [mm]	7.5	1.5

*HR_max_: The highest measured heart rate (beat·minute^−1^) during the protocol. $\dot{V}O_{2max}$: The highest 30-s moving average (based on 10-s mixing chamber values) during the incremental maximum test in the pretest*.

### Equipment

The protocol was performed on a 3-by-5-m motor-driven treadmill (ForceLink S-Mill, Motekforce Link, Amsterdam, the Netherlands) on roller skis with a friction coefficient of 0.016 [(see Seeberg et al. 2021) for details on the ski equipment and friction measurements].

Before testing, the body mass of each skier was determined on an electronic scale (Model No. 877, Seca GmbH and Co. Hamburg, Germany). Respiratory variables were measured continuously using open-circuit indirect calorimetry (Oxycon Pro, Erich Jaeger GmbH, Hochberg, Germany) and details of the setup and the calibration procedures are referred to in Seeberg et al. (2021). The data were collected as 10 s mixing chamber values and are presented as relative to body weight and as a percentage of $\dot{V}O_{2max}$ (%$\dot{V}O_{2max}$).

A Forerunner 920XT sports watch (Garmin Ltd., Olathe, KS, USA) was used to continuously measure HR at a sampling frequency of 1 Hz. Relative HR (%HR_max_) was calculated as the percentage of maximal HR (HR_max_) for each skier, and HR_max_ was the highest measured value for each person, which was obtained either in the HI session or in the protocol for determining $\dot{V}O_{2max}$ (protocol described in chapter 2.4). BLa was measured using a Biosen C-line Sport Lactate Measurement System (EKF Industrial Electronics, Magdeburg, Germany) after collecting 20 μl of blood from the fingertip. The device was calibrated every 60 min with a 12-mmol μl standard concentration. Rating of perceived exertion (RPE) for the upper body, lower body, and overall was recorded using the 6–20-point Borg Ratine of Perceived Exertion (RPE) Scale (Borg, 1982).

Muscle oxygenation was assessed using a wireless NIRS system (Portamon, Artinis Medical Systems, Elst, the Netherlands) consisting of two optodes [see details in Seeberg et al. (2021)]. Data from the two optodes was collected and synchronized in time by the designated software, and the tissue saturation index (TSI) with a fit factor exceeding 99.8% was used. Herein, TSI_leg_ indicates the TSI from the sensor placed on the vastus lateralis in the right leg, whereas TSI_arm_ is the TSI from the sensor placed on the long head of the triceps brachii in the right arm.

Eight Oqus 400 infrared cameras captured the 3D position of passive reflective markers placed bilaterally on the body, on roller skis, and on poles, all with a sampling frequency of 200 Hz using the Qualisys Pro Reflex system and Qualisys Track Manager software [Qualisys AB, Gothenburg, Sweden; details given in Seeberg et al. (2021)]. The motion capture system measured only every second lap (i.e., Laps 1, 3, 5, and 7) during LI and HI (the simulated mass start) to reduce the risk of data overload and system failure.

Instrumented ski pole grips (Proskida, Whitehorse, YT, Canada) were used to measure the axial (i.e., resultant) force directed along the poles. The data were streamed to a smartphone *via* Bluetooth and later downloaded to a computer and synchronized with the movement data. The sampling frequency of the force data was 100 Hz.

An IMU placed on the front of the chest (Physiolog 5, GaitUp SA, Lausanne, Switzerland) was used to provide continuous data of the motion of the upper body. The IMU consisted of a 3D accelerometer and 3D gyroscope with a sampling frequency of 256 Hz, in addition to a barometric pressure sensor with a sampling frequency of 64 Hz. The data were stored locally on the sensor and later downloaded to a computer. The movement of the skiers was also visually captured from behind with a video camera (GoPro Hero6, GoPro, Inc., San Mateo, CA, USA) also used to obtain the ground truth.

### Protocol for Determining $\dot{\text{V}}{\text{O}}_{2\max}$

The $\dot{V}O_{2max}$ measurements were taken a separate day before the primary data collection. The protocol of this day can be found in Noordhof et al. (2021). Briefly, the starting incline and speed were 10.5% and 11 km·h^−1^, respectively, after which the speed was kept constant, while the incline was subsequently increased by 1.5% every minute until 14.0%. Thereafter, the speed was increased by 1 km·h^−1^ every minute until exhaustion. The highest 30 s moving average, based on 10 s mixing chamber values, was defined as $\dot{V}O_{2max}$.

### Protocol

After attaching the wearable sensors to the body, the skiers stood still on the treadmill for 4 min while their baseline respiratory measurements were obtained. The active protocol began with a brief calibration procedure (including three jumps used to synchronize sensors from different sources and video) for the IMU sensor before the 18 min warm-up was performed at low to moderate intensity (5 min of G3 at 10 km·h^−1^ with a 5% incline) before two 4 min stages using G2 and G4 (10 km·h^−1^ at an 8% incline).

**Figure 1:**
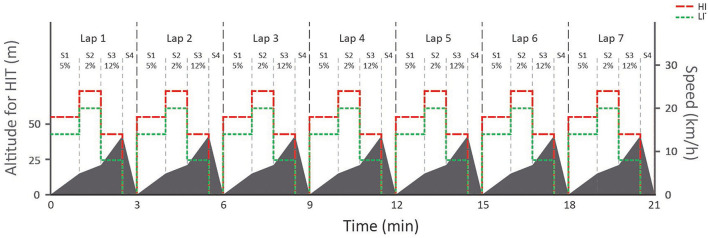
Protocol showing the speed of the treadmill for both the 21 min low-intensity (LI) and high-intensity HI sessions and changes in simulated altitude for the HI session. The course was divided into seven 3 min laps, each containing four segments (i.e., S1–S4) with the same inclines but different speeds for LI and HI.

The 21 min LI session followed the preset terrain profile shown in Figure 1. Thereafter, a 5 min recovery period was given before the 21 min HI session was performed on the same inclines but at higher speeds. The HI session was immediately followed by an incremental all-out sprint of which the data were not used in this study. The speeds in each segment during LI and HI were carefully selected based on pilot testing and the performance level of the participants. During the entire protocol, each skier received continuous visual and verbal feedback concerning the upcoming terrain and the time until the start of the next segment.

The track in the 21 min LI and HI sessions were organized into seven identical 3 min laps consisting of four different segments simulating a moderately uphill ascent (5%) (i.e., Segment 1), a flat segment (2%) (i.e., Segment 2), a steep uphill ascent (12%) (i.e., Segment 3), and a simulated downhill descent (i.e., Segment 4). The profile of the track was designed according to standards of the International Ski Federation (The International Ski Competition Rules ICR, 2020), in which the following sub-techniques are most commonly used (Andersson et al., 2010): Gear 2 (G2), a technique for skiing uphill (e.g., Segment 3) that involves a double-pole push in connection with every other leg push; Gear 3 (G3), a technique used on moderate inclines (e.g., Segment 1) and level terrain that involves a symmetrical pole push together with every leg push; Gear 4 (G4), a symmetrical double pole push in connection with every other leg push used on level terrain (e.g., Segment 2); Gear 5 (G5) only leg strokes are performed without poling; and Gear 7 (G7), used in a technique applied on downhill terrain (e.g., Segment 4) in which the skier is in a tucked position. In the simulated downhill, the skiers were holding a rope that was attached to the front of the treadmill while they were using a tuck position, thereby simulating G7. Although the track was designed for the use of specific sub-techniques on each segment, the skiers could freely select which sub-techniques they used.

### Data Analysis

#### Cycle Detection and Classification of Sub-techniques

The accelerometer data from the IMU placed on the chest were used to automatically detect and classify each individual cycle into a sub-technique using Gaussian filtering and a trained support vector machine learning model, following a method similar to what Rindal et al. (2018) used. Subsequently, the data were manually examined and corrected for errors in classification by comparing the classified cycles with the video and the graphical representation of filtered accelerometer signals. The accuracy of the model within those data exceeded 99%. Cycle detection was based on the sideways movements of the upper body, with the start of the cycle defined as the point at which the upper body was in a “left-position” with the lowest acceleration. Cycle detection and treadmill speed were used to derive the cycle length (CL) and cycle rate (CR) of each cycle. The cycles were classified into the sub-techniques G2, G3, G4, or other, the last of which included G5, transitions between sub-techniques, simulated downhill skiing (i.e., G7), and non-skiing activities. The algorithms for cycle detection, model development, and the classification of sub-techniques were implemented in MATLAB R2018b (MathWorks, Natick, MA, USA).

#### Determination of Pole and Ski Contact Time

Accelerometer and gyroscope data from the left and right wrists were used to calculate the contact time for poles (CT_pole_) by determining the time between initial and terminal pole contact with the ground. Initial contact was set as the first acceleration peak at the beginning of the vibration phase induced by the touching of the ground by the pole, while terminal contact was obtained as the highest acceleration peak close to the minimum angular speed induced by the change in direction of the motion to bring the poles back once the pole push was finished. Ski contact time (CT_ski_) was calculated using data from the IMUs mounted on the skis. Initial ski contact was defined as the first peak of the pitch angular velocity before a long phase with low angular velocity due to the skis being on the ground, while the terminal ski contact was set as the last negative vertical acceleration peak after the low angular velocity phase. The overall precision for CT_pole_ was 18 ± 21 ms (6.5 ± 11.5%) and for CT_ski_ was 7 ± 13 ms (0.7 ± 1.0%).

#### Absolute and Relative Pole and Ski Power

The center of mass (CoM) of the body was calculated based on the marker position data from the Oqus measurements [see (10) for details]. The mass properties of body segments were calculated according to de Leva (1996), while CoM velocity was determined by the numerical differentiation of the position data. In skate-style XC skiing, the instantaneous power is generated from poling and ski push-offs. Thus, pole power (P_pole_) was calculated from pole force (F_pole_) and CoM velocity (V_CoM_): [*P*_*pole*_ = *F*${}_{pole\text{\_}x}^{*}$V_*CoM*_*x*_ + $F_{pole\text{\_}y}^{*}$V_*CoM*_*y*_ + $F_{Pole\text{\_}z}^{*}$V_*CoM*_*z*_], with *x, y*, and *z* representing components of F_pole_ and V_CoM_ in the forward-backward (*x*), sideways (*y*), and vertical (*z*) directions (Donelan et al., 2002). P_pole_ was calculated independently for each pole and the values for each pole were subsequently summarized. The difference between work rate (P_cycle_) and average P_pole_ per cycle was interpreted as average ski power (P_ski_). Relative P_pole_ (%P_pole_) and relative P_ski_ (%P_ski_) were calculated as the percentage of the P_cycle_ for each skier, while relative P_poleleft_/P_polerigth_ (%P_poleleft_/%P_poleright_) was calculated as the percentage of the P_pole_ for each skier. One skier's power data for the entire test were missing due to technical issues and were therefore not included in the analysis.

#### Synchronization and Processing of Data

All continuously derived sensor data, namely, HR, $\dot{V}O_{2}$, TSI_leg_, TSI_arm_, CL, CR, sub-technique, CT_pole_, CT_ski_, P_cycle_, %P_pole_, %P_ski_, %P_poleleft_, and %P_poleright_ were synchronized to a common master timeline and compound into one dataset with a 1 Hz resolution before the means were calculated. Start and stop times for treadmill speed and incline, HR, $\dot{V}O_{2}$, and NIRS data were manually noted during the data collection, whereas the synchronization of IMU-derived data, namely, CL, CR, sub-technique, CT_pole_, and CT_ski_, were found by identifying three synchronization jumps from the calibration routine in the IMU data and on video. Reduction to the 1-Hz resolution was performed by calculating the mean for each second of data, except for the NIRS data, for which mean 1-Hz values were calculated over 2 s.

The terrain-dependent fluctuations (TDF) in HR, $\dot{V}O_{2}$, TSI_leg_, and TSI_arm_ were defined for each skier as


(1) $$\begin{array}{r} TDF=\;\frac{\sum_{Lap\;2}^{Lap\;7}\left(\%PeakVal-\%MinVal\right)}{NumLaps} \end{array}$$

where %PeakVal and %MinVal is given in % of max level and NumLaps is equal to 6 laps. Note that Lap 1 was excluded from these analyses since this lap starts from rest, which provides physiological values for this lap that deviate from the true fluctuation in metabolic demands.

Time-dependent change in the physiological variables (TDC_Phys_) from the start to the end of each session was quantified as


(2) $$\begin{array}{r} TDC_{Phys}=\%MeanVal\;Lap\;7-\%MeanVal\;Lap\;2 \end{array}$$

where %MeanVal is given in % of max level. Note that because Lap 1 starts directly from rest, Lap 2 was used instead of Lap 1.

For the biomechanical parameters, time-dependent change (TDC_BioMech_) was quantified as


(3) $$\begin{array}{r} TDC_{BioMech}=\;\%MeanVal\;Lap\;7-\%MeanVal\;Lap\;1 \end{array}$$

where %MeanVal is given in % of max level.

For one of the skiers, the treadmill stopped at Lap 7 at LI. Therefore, time-dependent changes were calculated without the data of that skier, as were other values of variables from Lap 7 for the skier.

The total distance and speed for LI/HI sessions were 5.8/7.5 km and 16.7/21.3 km/h, respectively. These numbers were calculated using the same speeds as in the protocol for Segments 1, 2, and 3; however, for Segment 4, the simulated downhill, 30 km·h^−1^ was used for LI and 35 km·h^−1^ for HI based on data from a previous field study (7) instead of the protocol-speed (20 km·h^−1^) to give more realistic numbers for total distance and average speed. Due to the safety of the skiers, the speed during the simulated downhill was intentionally kept lower than what is normally used in real downhills.

### Statistical Analysis

All data were tested for normality using the Shapiro–Wilk test in combination with the visual inspection of data and are presented herein as *M* ± *SD*.

A two-way repeated-measures analysis of variance was used to analyze the effect of segment and lap and their interactions on %HR, %$\dot{V}O_{2}$, TSI_arm_, TSI_leg_, and %P_pole_ at LI and HI separately. When significant primary effects were found, Tukey's *post-hoc* analysis was performed to determine pairwise comparisons.

For data that met the assumption of normality, mean values for all variables at LI and HI sessions, including terrain-based fluctuations, biomechanical variables, and power variables for separate sub-techniques both for the entire sessions and in segments, were compared using a paired sample *t*-test (Table 2 and Supplementary Tables 1, 2). TSI_arm_/TSI_leg_, CT_pole_/CT_ski_, %P_pole_/%P_ski_, and terrain-based fluctuations in %HR and $\%\dot{V}O_{2}$ were compared using paired *t*-tests separately for LI and HI data. Non-normally distributed data (i.e., only P_poleright_ in G4 and P_poleleft_ in G4, shown in Supplementary Table 1) were compared using a non-parametric Wilcoxon signed-rank test.

**Table 2:** Heart rate (HR), oxygen uptake ($\dot{V}O_{2}$), tissue saturation index (TSI) for the leg (TSI_leg_) and arm (TSI_arm_), power variables, rate of perceived exertion (RPE), and blood lactate (BLa) for the low-intensity (LI) vs. high-intensity (HI) sessions, with means (*M*), standard deviations (*SD*), and *p*-values.

		**LI**		**HI**			**HI–LI**
**Period: Laps 1–7**	***N***	***M***	***SD***	***M***	***SD***	***p***	**Δ (%Δ)**
Mean %HR [in % of HR_max_]	9	74.1	3.1	89.7	2.5	<0.01	15.5 pp
Mean %$\dot{V}O_{2}$ [in % of $\dot{V}O_{2max}$]	9	58.3	2.4	79.4	3.5	<0.01	21.0 pp
Mean $\dot{V}O_{2}$ [mL/kg/min]	9	41.1	0.8	55.9	1.7	<0.01	14.8 (36%)
Mean TSI_leg_ [%]	9	64.6	3.8	58.3	6.7	<0.01	−6.3 pp
Mean TSI_arm_ [%]	9	52.6	7.1	48.8	9.0	0.03	−3.8 pp
Whole-body RPE	9	12.7	1.2	17.2	1.5	<0.01	4.5 (35%)
Upper-body RPE	9	12.6	1.2	17.3	1.7	<0.01	4.7 (37%)
Lower-body RPE	9	12.8	1.2	17.2	1.5	<0.01	4.4 (34%)
BLa [mmol/L]	9	1.3	0.3	11.1^**^	2.1	<0.01	9.8 pp
Peak %HR [in % of HR_Max_]	9	81.4	3.4	98.4	2.0	<0.01	17.0 pp
Peak %$\dot{V}O_{2}*$ [in % of $\dot{V}O_{2max}$]	9	75.9	4.9	98.6	3.2	<0.01	22.7 pp
%HR fluctuation [pp]	9	17.7	2.7	12.2	3.3	<0.01	−5.3 pp
%$\dot{V}O_{2}$ fluctuation [pp]	9	31.7	4.4	33.0	1.7	0.50	1.3 pp
%TSI_leg_ fluctuation [pp]	9	12.6	3.3	24.3	9.3	<0.01	11.7 pp
%TSI_arm_ fluctuation [pp]	9	23.9	6.1	33.4	13.9	0.04	9.5 pp
**Period: Laps 1, 3, 5, and 7**		**LI**		**HI**			
P_cycle_ [Watt]	8	205	15	291	21	<0.01	85.9 (42%)
%P_pole_ [in % of P_cycle_]	8	55.7	5.1	57.6	5.2	0.31	1.9 pp
%P_ski_ [in % of P_cycle_]	8	44.3	5.1	42.4	5.2	0.31	−1.9 pp
%P_poleleft_ [in % of P_pole_]	8	47.9	5.6	47.6	3.5	0.88	−0.3 pp
%P_polerigth_ [in % of P_pole_]	8	52.0	5.0	51.3	3.6	0.88	−0.7 pp
**Change: Lap 7–Lap 2**		**LI**		**HI**			
%HR [in % of HR_max_]	8	2.3	2.0	7.8^*^	1.1	<0.01	5.5 pp
%$\dot{V}O_{2}$ [in % of $\dot{V}O_{2max}$]	8	−0.4	0.7	1.1	2.7	0.34	1.5 pp
TSI_leg_ [%]	8	−2.1	1.6	−1.2	2.4	0.53	0.8 pp
TSI_arm_ [%]	8	−0.6	1.1	−2.9	2.0	0.05	−2.4 pp
**Change: Lap 7–Lap 1**							
%P_pole_ [in % of P_cycle_]	7	−6.6^*^	2.5	−7.7^*^	4.5	0.48	−1.1 pp
%P_ski_ [in % of P_cycle_]	7	6.6^*^	2.5	7.7^*^	4.5	0.48	1.1 pp
%P_poleleft_ [in % of P_pole_]	7	−0.4	1.5	0.5	1.5	0.24	0.9 pp
%P_polerigth_ [in % of P_pole_]	7	0.4	1.5	−0.5	1.5	0.24	−0.9 pp

*The time-dependent change from Lap 2 to Lap 7 for HR, $\dot{V}O_{2}$, and TSI variables and from Lap 1 to Lap 7 for power variables are shown as well*.

*Note. pp = percentage points. HR_max_ = the highest heart rate (beats per minute) measured during the protocol. $\dot{V}O_{2max}=$ the highest 30-s moving average (based on 10-s mixing chamber values) during the maximal incremental test, Δ = difference in mean value between HI and LI, %Δ = percentage of difference in mean value between HI and LI relative to LI, P_cycle_ = mean power for cycle, %P_pole_ = relative power from poling, %P_ski_ = relative power from ski push-offs, %P_poleleft_ = relative power from left pole, %P_polerigth_ = relative power from right pole, %HR/%$\dot{V}O_{2}$/%TSI fluctuation = mean value of the difference between maximum and minimum values for each lap, with Lap 1 excluded due to starting from rest. Non-significant numbers are highlighted in light gray*.

* *Significant time-dependent change (p <0.05): HR, $\dot{V}O_{2}$, TSI_arm_, and TSI_leg_ Lap 7–Lap 2, power: Lap 7–Lap 1*.

** *BLa was measured after HI and the all-out sprint*.

A two-way repeated-measures analysis of variance was used to analyze the effect of intensity on time-dependent changes from Lap 1 (i.e., biomechanical and power) or Lap 2 (i.e., physiological) with Lap 7 and their interactions. If main effects were found, pairwise comparisons were done with a paired sample *t*-test (Table 2 and Supplementary Table 1).

The level of statistical significance was set at α = 0.05. All statistical analyses were performed using SPSS version 26.0 (SPSS Inc., Chicago, IL, USA).

## Results

**Figure 2:**
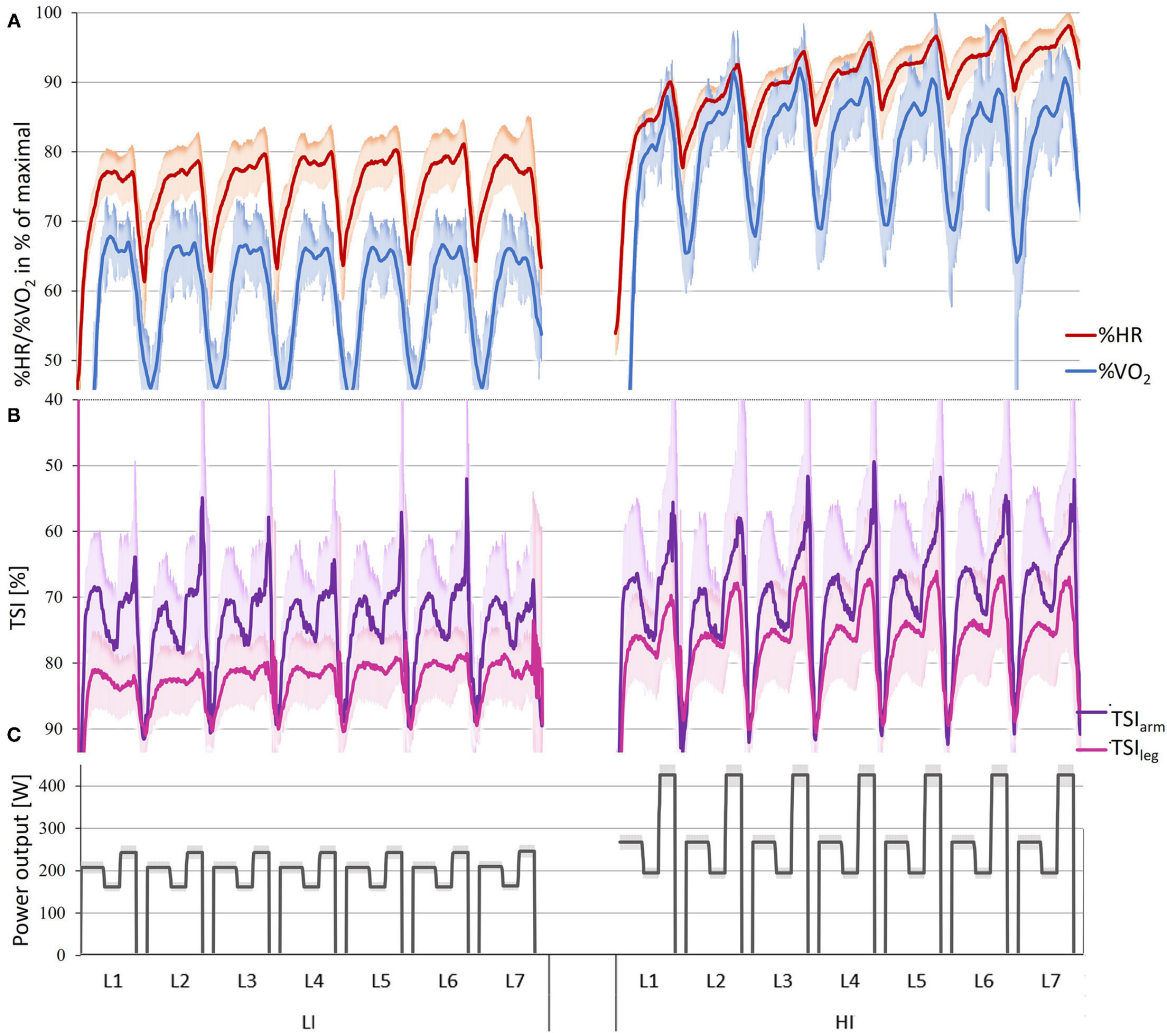
**(A)** Relative heart rate (%HR) and relative oxygen uptake (%$\dot{V}O_{2}$ 30 s moving average), **(B)** tissue saturation index (TSI) for the vastus lateralis in the right leg (TSI_leg_ and the long head of the triceps brachii in the right arm (TSI_arm_), and **(C)** power output for the LI and HI sessions (*M* ± *SD*) with 1-Hz resolution. For TSI, the vertical axis is reversed.

**Figure 3:**
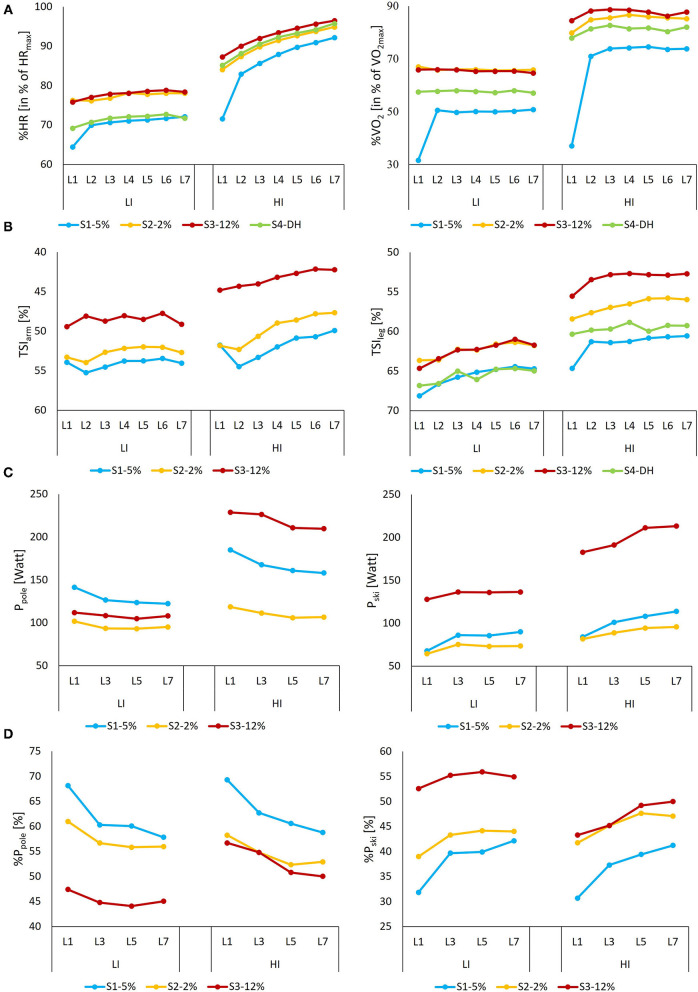
**(A)** %HR and %$\dot{V}O_{2}$, **(B)** TSI_arm_ and TSI_leg_, **(C)** absolute and **(D)** relative power for pooling (P_pole_ and ski push-offs (P_ski_ for each segment (S1–S4) as a function of the lap (i.e., L1–L7) during LI and HI sessions. For TSI, the vertical axis is reversed, and TSI_arm_ in the simulated downhill segment (i.e., S4) is not shown due to excessive movement noise in the signal when the skiers grabbed the rope.

**Figure 4:**
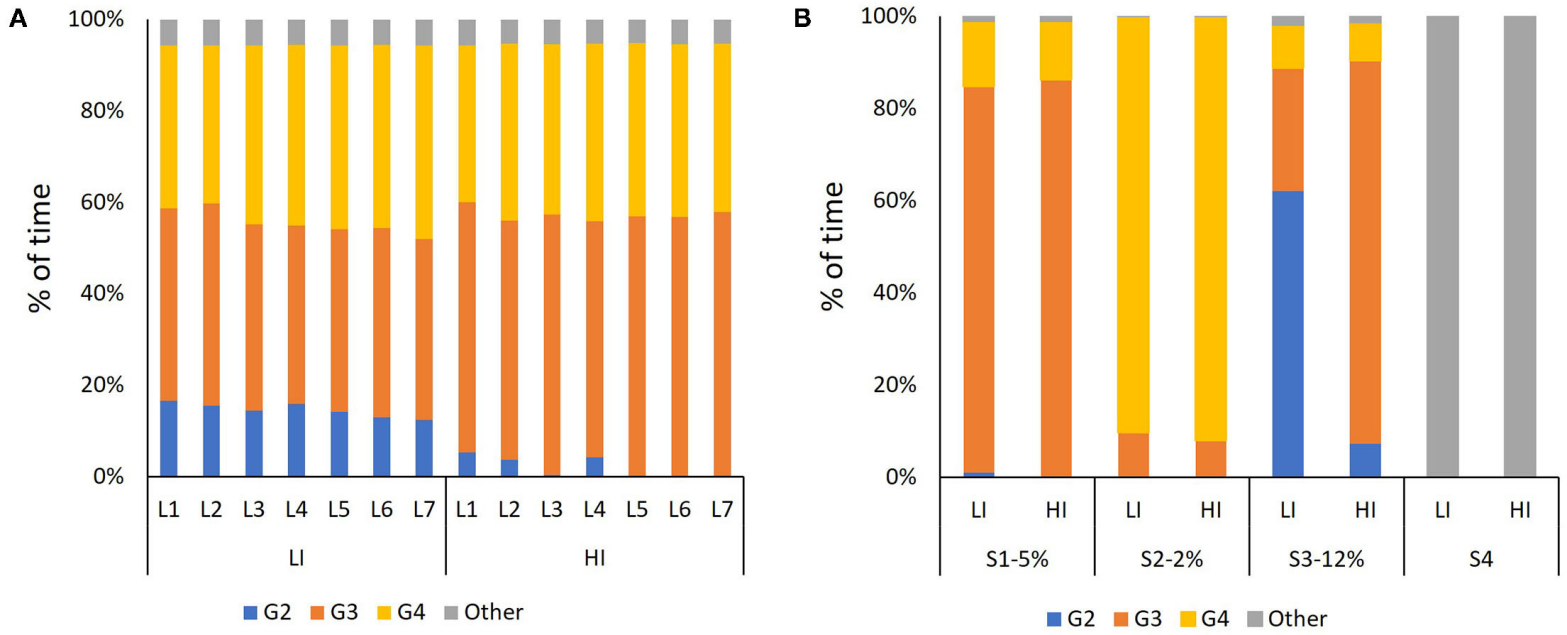
Distribution of sub-techniques, in the percentage of the time, **(A)** as a function of the lap (i.e., L1–L7) and **(B)** as a function of segment (i.e., S1–S5), in the LI and HI sessions.

**Figure 5:**
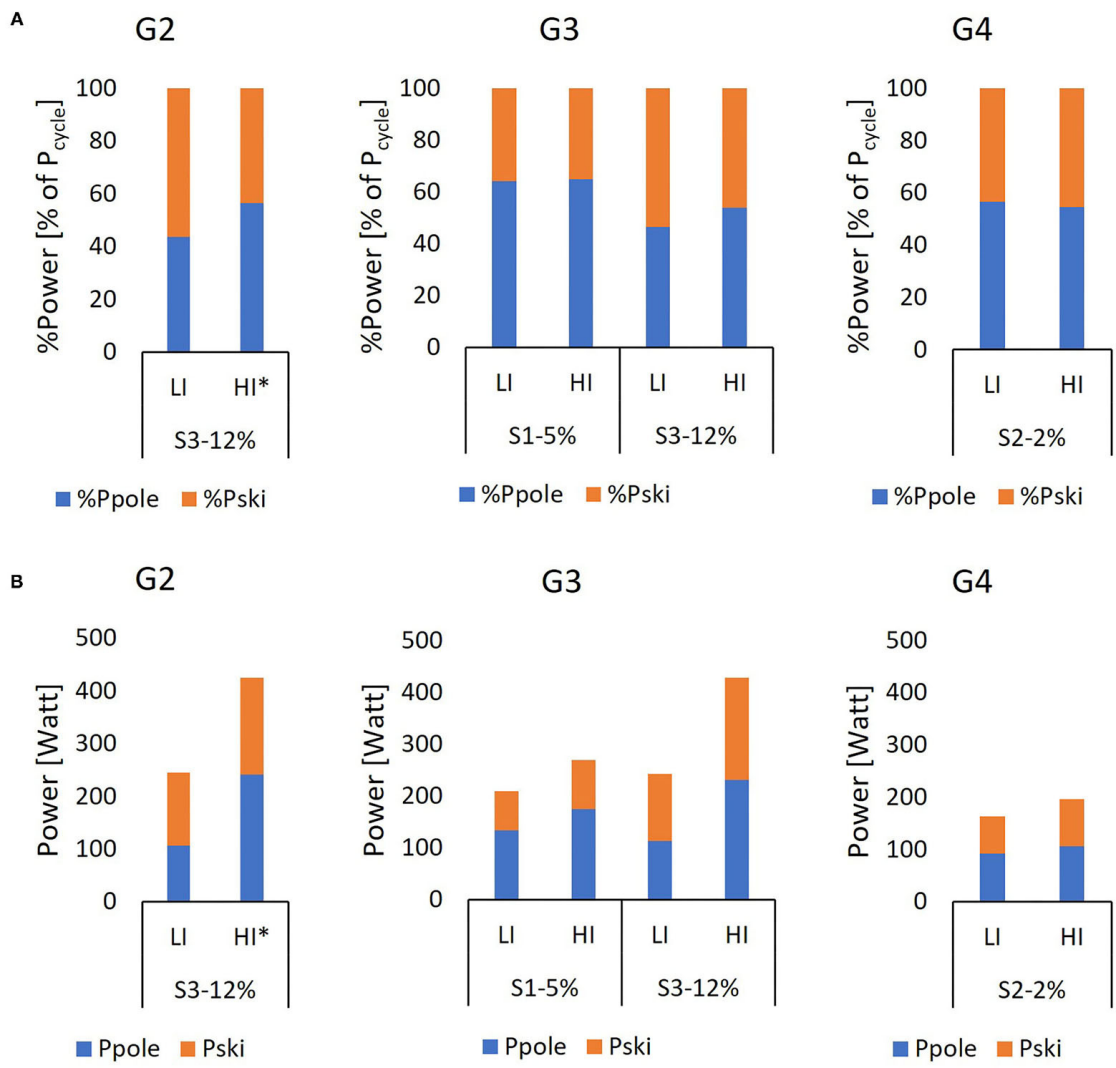
Mean values for **(A)** relative and **(B)** absolute pole power (%P_pole_, P_pole_) and ski power (%P_ski_, P_ski_) during the LI and HI sessions for all sub-techniques and segments (i.e., S1–S3) for all skiers (*n* = 8) with power data. During the HI session, G2 was used only by five skiers, one of whom was missing power data, hence the small sample.

In the following sections, the results are presented from three different levels: fluctuations of variables with 1 s resolution (Figure 2), overall means for the entire 21 min LI and HI sessions divided and not divided into different sub-techniques (Table 2 and Supplementary Table 1), and means for laps and segments (Figures 3–**5** and Supplementary Table 2).

### Physiological Responses

Fluctuations in physiological variables for LI and HI according to the set inclines and workloads in LI and HI are shown in Figure 2, while values averaged overlaps and segments for the same variables are displayed in Figure 3.

Significant interaction effects between lap and segment were found at both LI and HI for $\%\dot{V}O_{2}$ and at HI for %HR (all *p* < . 01) but not at LI for %HR (*p* = 0.88). At both LI and HI, significant main effects of segment occurred for both %HR and %$\dot{V}O_{2}$ (all *p* < 0.01), as shown in Figure 3A. At LI, %HR and %$\dot{V}O_{2}$ did not differ between Segments 2 (2% uphill) and 3 (12% uphill) (%HR: *p* = 0.88, %$\dot{V}O_{2}$: *p* = 0.83) and were higher than in the other segments (all *p* < 0.01). In addition, mean values for Segments 1 (5% uphill) and 4 (downhill) did not differ for %HR (*p* = 0.15), while %$\dot{V}O_{2}$ for Segment 1 was lower than for Segment 4 (*p* < 0.01). At HI, Segments 2 and 4 did not differ in %HR (*p* = 0.36), which was higher than in Segment 1 and lower than in Segment 3 (both *p* < 0.01). Concerning %$\dot{V}O_{2}$, however, all segments differed (all *p* < 0.01). For %HR, interaction effects between intensity and time-dependent changes (from Lap 2 to Lap 7) as well as effects for intensity and time-dependent time were found (all *p* < 0.01), while for %$\dot{V}O_{2}$, significant effects were only found for intensity (*p* < 0.01). At LI, a significant main effect of the lap was found for %$\dot{V}O_{2}$ and %HR (both *p* < 0.01). However, only Lap 1 differed significantly from the other laps (all *p* < 0.01), for it began from the rest (all other *p* > 0.30). At HI, both %$\dot{V}O_{2}$ and %HR had significant main effects in relation to lap (both *p* < 0.01). Only in Lap 1 did %$\dot{V}O_{2}\;$significantly differ from the other laps. However, for %HR, HR slowly increased toward the end of the session (Table 2).

At LI and HI, no significant interaction effects between lap and segment were found for TSI_arm_ or TSI_leg_ (all *p* = 1.00). However, significant main effects of the segment were found for both variables (*p* < 0.01) (Figure 3B). For neither TSI_arm_ nor TSI_leg_, significant main effects were not found between intensity and time-dependent changes from Lap 2 to Lap 7 (all *p* > 0.57). Although no significant main effect of the lap was found for TSI_arm_ at LI or HI or for TSI_leg_ at HI (*p* = 1.00), a significant main effect of the lap in Lap 1 was found for TSI_leg_ (*p* = 0.04), which during Lap 6 was less than during Lap 1 (*p* = 0.05).

Mean physiological variables at LI and HI are shown in Table 2. Due to the higher workload at HI than at LI, %HR, %$\dot{V}O_{2}$, RPE and BLa were higher at HI while TSI_arm_ and TSI_leg_ were lower. Terrain-based fluctuations in %VO_2_ did not differ between HI and LI (~32%-points, *p* = 0.50). By comparison, %HR fluctuated less than %$\dot{V}O_{2}$ at HI than at LI (-12.2%-points, *p* < 0.01) and displayed a time-dependent increase at HI (7.8%-points, *p* > 0.01). Both TSI_arm_ and TSI_leg_ terrain-based fluctuations were greater at HI than LI (TSI_arm_: 9.5%-points, TSI_leg_: 11.7%-points, both *p* < 0.01). Compared with TSI_leg_, TSI_arm_ had high terrain-based fluctuations (LI: 11.3%-points, HI: 9.1%-points, *p* < 0.05) and low mean values both at LI (-12%-points, *p* < 0.01) and HI (-9.5%-points, *p* < 0.01). However, TSI_leg_ decreased more than TSI_arm_ (2.4%-points, *p*= 0.05) from LI to HI.

### Sub-technique Selection and Biomechanical Responses

**Figure 6:**
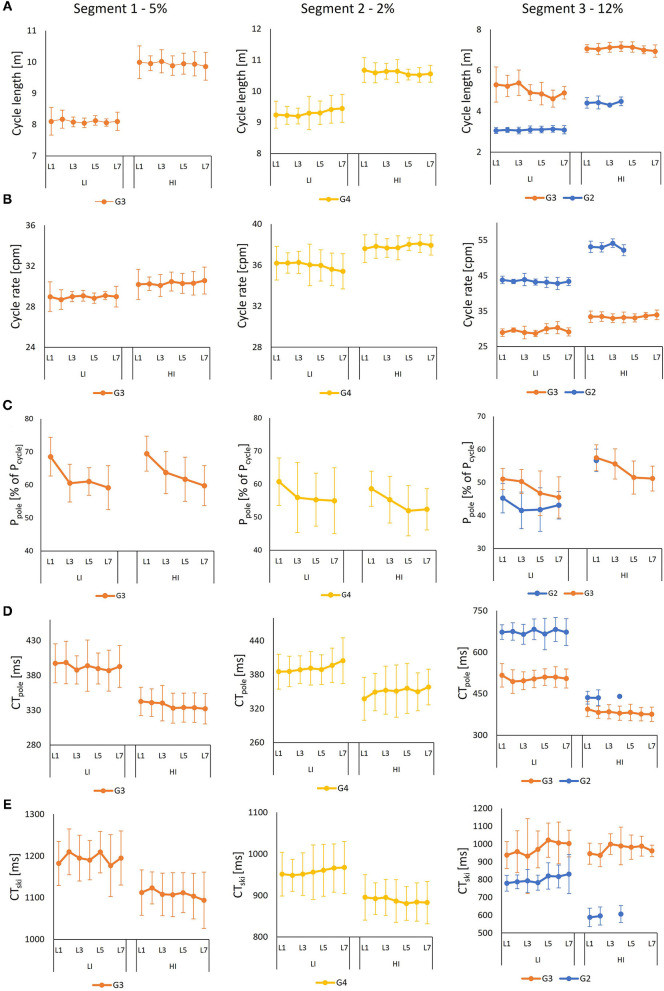
**(A)** Cycle length (CL), **(B)** cycle rate (CR, in cycles per minute, cpm), **(C)** relative power contribution from pooling (in % of total power for each cycle), **(D)** contact time for pole (CT_pole_), and **(E)** contact time for ski (CT_ski_), all variables as a function of the lap (i.e., L1–L7) during the LI and HI sessions for each segment (*M* ± *SD*). Power from ski push-offs is given as %P_ski_ = 100%–%P_pole_. Power contribution was measured only for odd lap numbers and was not available for one of the skiers. If the skier spent <6 s on a particular sub-technique in one segment or lap, then it was excluded from the analysis. During the HI sessions, G2 was used only by five skiers, one of whom was missing power data, hence the small sample.

The selection of sub-techniques during the different laps and segments at LI and HI is displayed in Figure 4, while corresponding temporal patterns as a function of a sub-technique, segment, and/or lap at both LI and HI are provided in **Figure 6** and Supplementary Tables 1, 2.

At LI, the skiers primarily selected G3 during Segment 1, G4 during Segment 2, and G2 during Segment 3 (Figure 4B and Supplementary Table 1). At HI, the same pattern emerged, with the primary difference being the greater use of G3 during Segment 3 (*p* < 0.05). Neither CL nor CR changed significantly from Lap 1 to Lap 7 for any of the sub-techniques (all *p* > 0.05), as shown in Figure 5. For all sub-techniques, CL was longer and CR was higher at HI than at LI (Figures 6A,B and Supplementary Tables 1, 2). The relative change from LI to HI was greater for CL than for CR in both overall (Supplementary Table 1) and by segment (Supplementary Table 2).

At both LI and HI, CT_pole_, CT_ski_, %CT_pole_, and %CT_ski_ depended on the used sub-technique and segment, with CT_pole_ being shorter than CT_ski_ for all sub-techniques (Figures 6D,E and Supplementary Tables 1, 2). At HI during G4, CT_pole_ showed a time-dependent change from Lap 1 to Lap 7, with a 6.7% longer poling time (*p* < 0.01). For the other sub-techniques and CT_ski_, no significant change arose (all *p* > 0.05).

Overall, CT_pole_, CT_ski_, and %CT_pole_ were lower for all sub-techniques at LI than at HI (Supplementary Table 1). However, there was no change in %CT_ski_ between G2 and G3 (Supplementary Table 1). When divided into segments, both CT_pole_, CT_ski_, %CT_ski_, and %CT_ski_ were lower at LI than at HI in all cases except for %CT_ski_ during G2 in Segment 3 (Supplementary Table 2). Added to that, for HI, CT_pole_ was shorter than CT_ski_ for all sub-techniques (Supplementary Tables 1, 2).

### Power Distribution

The relative and absolute ski and pole power during each segment and sub-technique are displayed in Figure 5, with the relative power for each segment as a function of lap number shown in Figure 6C.

At LI and HI, no significant interaction effects between lap and segment were found in overall %P_pole_ or %P_ski_ (= 100%–%P_pole_) (both *p* > 0.86). However, significant main effects were found for segment (both LI and HI *p* < 0.01). Beyond that, %P_pole_ in Segments 1 and 2 at LI did not differ (*p* = 0.69) and was higher than in Segment 3 (all *p* < 0.01), while at HI %P_pole_ in Segments 2 and 3 did not differ (*p* = 0.61) and was less than in Segment 1 (all *p* < 0.01). In addition, %P_pole_ was significantly higher than %P_ski_ (11.5%-points, *p* = 0.02). No significant main effects were found for %P_pole_ and %P_ski_ between intensity and time-dependent changes from Lap 2 to Lap 7 (*p* = 0.69). However, significant effects were found for the time-dependent changes (*p* < 0.01). At both LI and HI, a significant main effect of the lap was found for %P_pole_, with a time-dependent change toward relatively more power from the ski push-offs (i.e., Lap 7 – Lap 1), both overall (*p* < 0.01, Table 2), and for G3 and G4 separately (*p* < 0.01, Figure 5A).

Overall mean values for %P_pole_ and %P_ski_ did not differ between LI and HI (Table 2), and %P_pole_ was significantly higher than %P_ski_ at both LI (11.5%-points, *p* = 0.02) and HI (15.2 %-points *p* < 0.01). %P_pole_ depended on sub-technique and segment (Figure 5A and Supplementary Tables 1, 2).

The relative contribution of power from the right and left poles differed between individuals independent of sub-technique and incline and did not show the main effects of laps during any sub-techniques at LI or HI (*p* > 0.05), as shown in Table 2 and Supplementary Table 1.

## Discussion

The primary purposes of our study were to investigate physiological and biomechanical responses to LI and HI roller ski skating on varying terrain and to compare the responses between training intensities. Here, we provide a novel understanding of the complex physiological and biomechanical stimuli provided by LI and HI training in XC skiing by highlighting three primary findings. First, both LI and HI training on varying terrain induced large terrain-dependent fluctuations in HR, $\dot{V}O_{2}$, and muscle oxygen saturation. In addition, the power distribution generated by poles and skis depended on the sub-technique employed both for LI and HI, with a time-dependent shift toward gradually more power coming from the ski push-off from the start to the end of each session within all sub-techniques. Second, terrain-based fluctuations in $\dot{V}O_{2}$ were similar at LI and HI, whereas HR fluctuated less at HI and demonstrated a time-dependent increase in HR. The arm muscle vs. leg muscle oxygen saturation ratio changed, and an interaction effect arose between segments amid increased intensity from LI to HI. Third, the G2 sub-technique was employed more than G3 on the steepest uphill section at LI than at HI, while CL increased two to three times more than CR, and CT_pole_ decreased more than CT_ski_ from LI to HI when compared in the same terrain.

Both the LI and HI sessions were performed on varying terrain and induced terrain-dependent fluctuations in %HR and %$\dot{V}O_{2}$, thereby exemplifying the interval-based cardiovascular and muscular loading during XC skiing. This clearly differs from training at similar intensities in most other exercise modalities or endurance sports in which loading is more stable (Sandbakk et al., 2021). The simultaneous measurements of %$\dot{V}O_{2}$ and %HR show that the timing of the peak values (i.e., in or after the steepest uphill ascents) of %$\dot{V}O_{2}$/%HR were independent of intensity. However, the decrease in %HR during the simulated downhill segment was less and delayed compared with the larger (and faster-responding) decrease in %$\dot{V}O_{2}$. Those differences also depended on intensity, with the decrease in %HR during downhills in HI being less than at LI, thereby confirming the results of several other studies (Gløersen et al., 2018; Karlsson et al., 2018; Solli et al., 2018; Haugnes et al., 2019). Such results can be explained by skiers often driving the intensity up to and above $\dot{V}O_{2max}$ in uphill ascents. Indeed, in our study, the skiers reached 98.4% of HR_max_ (range: 94.3–100%) and 98.6% of $\dot{V}O_{2}\text{max}$ (range: 93.2–102.1%), and the subsequent oxygen deficit resulted in a reduced and delayed HR recovery (Gløersen et al., 2020). The significant time-dependent change in %HR at HI also differed from the %$\dot{V}O_{2}$ response, which remained stable throughout the LI and HI sessions except from Lap 1, at which the %$\dot{V}O_{2}$ was lower due to the starting of skiers from rest. Altogether, those findings have important implications for the interpretation of %HR during training and competitions, which is often used as a real-time proxy for %$\dot{V}O_{2}$ during non-steady-state exercises such as XC skiing (Solli et al., 2018; Haugnes et al., 2019). Thus, the degree to which %HR can be used to accurately indicate %$\dot{V}O_{2}$ during interval-like or even continuous exercise clearly seems to depend on duration, intensity, and fluctuations in intensity (Boulay et al., 1997; Bot and Hollander, 2000; Tucker et al., 2006; Kolsung et al., 2020). Since HR has limitations in its ability to reflect metabolic intensity in XC skiing, a practical solution may be to complement HR measures with perceived exertion, in addition to analyses of blood lactate concentration on selected sessions, to decide exercise intensity during training.

Complementary to HR and $\dot{V}O_{2}$ measurements, muscle oxygen saturation, measured by the TSI in the arms (triceps brachii) and legs (vastus lateralis), provide valuable indications about the local metabolism of the working muscles. Similarly, the TSI-values also fluctuated according to the terrain both at LI and HI, with only slight delays in kinetics. Similar fluctuations in muscle oxygen saturation during interval-like HI training were found when oxygen saturation in working muscles (i.e., biceps brachii, triceps brachii, latissimus dorsi, and vastus lateralis) was measured during successive upper-body sprints (Sandbakk et al., 2015a). In our study, TSI values for both arms and legs decreased significantly from LI to HI. However, the terrain-dependent fluctuations in oxygen saturation in the arms differed from the corresponding measurements in the legs and were not associated with the amount of power generated by the arms vs. legs (Figures 3B–D). That finding aligns with results from a recent case study during a long-term competition in double poling, in which TSI measures for the triceps brachii showed larger terrain-based fluctuations than for the vastus lateralis (Stöggl and Born, 2021). Our findings thereby indicate that the desaturation of the muscles depends more on whole-body stress (i.e., %HR and %$\dot{V}O_{2}$) than the contribution from specific muscles, as previously suggested (Im et al., 2001). However, studies focusing more specifically on this issue are needed to conclude.

Mean TSI_arm_ was less than mean TSI_leg_ at both LI and HI, which indicates less oxygen saturation in the arms than the legs at both intensities. That finding aligns with results from two other studies in which elite skiers performed diagonal stride (Björklund et al., 2010) and double poling (Stöggl et al., 2013). Interestingly, the mean value for TSI_leg_ decreased more than that for TSI_arm_ from LI to HI, thereby indicating that for XC skiing the muscular load of the arms seems to be more independent of the overall internal and external intensity than the muscular load of the legs. That finding may have implications for understanding the specific muscular workload of LI training on varying terrain, by indicating that skiers can experience a high muscular training load in the arms at LI as well as at HI.

For both LI and HI, the skiers adapt their technique to the workload by generating more power from poling than ski push-offs, which is in line with the TSI measurements showing that TSI_arm_ was lower than TSI_leg_ at both LI and HI. Also, the distribution of pole and ski power seems less dependent on intensity than on sub-technique and thus incline. At LI, for example, we found more ski than pole power in the steep uphill ascent, both in G2 and G3, whereas more pole power was produced in G3 during the moderate incline. In addition, the findings related to G3, with more pole power produced at lower inclines, align with results from a study of the distribution of power generated by the arms and legs during double poling (Danielsen et al., 2019), in which the authors found that double poling at a 12% incline required less power from the arms than at a 5% incline, partly due to less advantageous working conditions for the arms with shorter poling times and a reduced angle between the arms and the ground at steeper uphill's. Because G3 and double poling have synchronized, highly similar arm movements, the same could be assumed to apply to our findings; that is, that less advantageous working conditions for the arms are causing the reduced power contribution from the arms at higher inclines. However, those aspects require further examination by using a specifically designed experimental setup.

Independent of intensity and sub-technique, the relative pole and ski power distribution gradually changed, with a higher contribution of ski power toward the end of the session. The change in power distribution could have been done intentionally to save the legs toward the end, or else because the skiers became more fatigued in the upper than in the lower body and therefore gradually generated more ski power. That compromise between generating arm (i.e., pole) and leg (i.e., ski) propulsion during skiing likely depends on individual resources and on how skiers pace their arms and legs, an aspect that requires more attention in future research. However, the change in power distribution did not influence CL or CR, which varied according to incline, speed, and sub-technique used but showed the same pattern within each sub-technique for all laps.

At LI, the skiers primarily selected G3 in the moderately uphill segment, G4 in the flat terrain, and G2 in the steep uphill ascent. HI had a similar pattern in sub-technique distribution as LI, with the exception of the steepest uphill (12% incline), where the skiers used more G3 and less G2 at HI than at LI. Accordingly, our findings indicate that skiers apply the same sub-techniques independently of training intensity across flat, and moderately uphill terrain, which is also the case for downhill terrain. A practical implementation of that finding could be to perform LI sessions on less strenuous terrain to enable the use of the same sub-techniques used during HI sessions with relatively little effort. Furthermore, it seems important to prioritize training in G3 at high speeds during steep uphill ascents as part of HI or sprint sessions, because that skill is more challenging to practice at LI.

Both macro and micro-kinematic variables depended on sub-technique and incline, as previously found in other studies (Nilsson et al., 2004; Stöggl and Müller, 2009; Sandbakk et al., 2012). CL and CR increased with higher intensity and speed, with CL increasing 2–3 times more than CR from LI to HI in most cases. That observation contrasts with past observations, in which CR has been identified as the primary driver of speed at moderate to high speeds (Nilsson et al., 2004; Stöggl and Müller, 2009), but agrees with other findings that both CR and CL did increase with speed (Sandbakk et al., 2012, 2015b). A practical takeaway from our findings is that skiers, to simulate competition-relevant CLs could include periods in their LI training during which they intentionally aim to ski with a lower CR than normal, as done in other sports, including road cycling (Aasvold et al., 2019). Such low-frequency training may be particularly relevant in relatively flat (or gentle downhill) terrain where CL has been shown as the main driver of increased speed.

Coinciding with the increase in CL and CR with speed, CT_pole_ and CT_ski_ decreased from LI to HI within all sub-techniques. Although that decrease in CT_pole_ is natural because CT_pole_ is highly dictated by speed, CT_ski_ can be maintained at a higher speed by angling the skis forward. However, in future studies, CT_ski_ should be divided into push-off and gliding (i.e., no push-off) time, where it would be expected that glide time increase with speed while ski push-off time remains more constant, in order to provide a more nuanced understanding of how this variable change with intensity. For both poles and skis, %CT_pole_ and %CT_ski_ also decreased from LI to HI. However, that change was far smaller than the change in CT_pole_ and CT_ski_. Although the power output of skiers is higher, skiers are forced to produce the power over shorter periods, with longer relative recovery times within a cycle, at higher intensities. That trend means altered muscle contraction dynamics and requires the ability to produce high power over a short time.

## Strengths and Limitations

The advantage of performing our study indoors while participants roller skied on a treadmill was that both physiological and biomechanical variables could be measured more accurately than while outdoors on snow. However, the differences between our setup and real-life situations when skiing outdoors require interpreting our results with caution. In addition, speed was preset for each incline at LI and HI, and even though the incline–speed combinations were carefully selected, the results could have differed if the skiers had freely chosen their speeds. Accordingly, the design of our study allowed us to investigate the underlying physiological and biomechanical mechanisms while skiing at LI and HI and thereby increase the generalizability of our results. A tradeoff, however, was a limitation in ecological validity. Even so, our protocol reflects the reality of elite skiers, who often perform LI and HI training together in groups.

## Conclusions

Both LI and HI XC skiing on varying terrain induce large terrain-dependent physiological and biomechanical fluctuations, similar to the patterns found during HI XC skiing. The primary differences between training intensities were the time-dependent increase in HR, reduced relative oxygen saturation in the legs compared to the arms, and the greater use of G3 on steep uphill terrain at HI, whereas sub-technique selection, CR, and pole vs. ski power distribution were similar across intensities on flat and moderately uphill terrain. In addition, the distribution between ski and pole power, including the gradual time shift toward more ski power from the start to the end of each session, seemed to depend more on sub-technique and incline than intensity. Overall, our findings illustrate unique physiological and biomechanical responses when XC skiing in varying terrain that coaches and athletes should be aware of when planning LI and HI endurance training. Accordingly, coaches should carefully choose intensity and terrain based on the specific development goal for each session. For example, it might be beneficial to prioritize less strenuous terrain during LI sessions to enable the employment of similar sub-techniques but with less effort than for HI sessions. Beyond that, the findings provide a good starting point for future studies, both for delving deeper into those mechanisms and opens for more applied approaches performed outdoors in future studies.

## Data Availability Statement

The original contributions presented in the study are included in the article/Supplementary Materials, further inquiries can be directed to the corresponding author/s.

## Ethics Statement

Ethical review and approval was not required for the study on human participants in accordance with the local legislation and institutional requirements. The patients/participants provided their written informed consent to participate in this study.

## Author Contributions

DN, JD, and KS conducted data collection. TS, ØS, and JK prepared the manuscript. JD calculated power distribution. JK synchronized and facilitated the sensor data. FM developed the model for extracting CT_pole_ and CT_ski_. ØS provided expert knowledge in the field and was responsible for designing the experiment. TS developed the model for cycle detection and sub-technique classification, explored and analyzed the data, constructed figures and tables, and was responsible, together with ØS, for finalizing the manuscript. All authors contributed to the overall concepts, protocol, sensor setup, and framework presented in the manuscript, as well as to its revision and ultimate approval.

## Funding

This study was supported by the AutoActive project (Project No. 270791), a research project in the IKTPLUSS program financed by the Norwegian Research Council.

## Conflict of Interest

The authors declare that the research was conducted in the absence of any commercial or financial relationships that could be construed as a potential conflict of interest.

## Publisher's Note

All claims expressed in this article are solely those of the authors and do not necessarily represent those of their affiliated organizations, or those of the publisher, the editors and the reviewers. Any product that may be evaluated in this article, or claim that may be made by its manufacturer, is not guaranteed or endorsed by the publisher.
