# Supplementary material for: Physiological and Biomechanical Responses to Cross-Country Skiing in Varying Terrain: Low- vs. High-Intensity
Source: Front Physiol. 2021 Oct 11;12:741573. doi: 10.3389/fphys.2021.741573 (PMC8543002; doi:10.3389/fphys.2021.741573)
Supplement: Supplementary file 1 [file Table_1.docx]

**Supplementary Table 1** Selection of sub-techniques (in % of time), temporal patterns in form of cycle length (CL), cycle rate (CR), cycle time (CyT), contact time for poles (CT_ski_) and skis (CT_ski_), and power variables for the low-intensity (LI) versus high-intensity (HI) sessions, with means (*M*), standard deviations (*SD*), and *p* values for all laps (only Laps 1, 3, 5, and 7 for power distribution) for all segments together. The sub-techniques displayed are G2, G3, and G4; the rest of the cycles were categorized as “Other” (LI: 19.8% ± 0.8, HI: 19.8% ± 1.2). Data for skiers who spent less than 6 s on a sub-technique were disregarded.

|  |  | **G2** | | | | | | **G3** | | | | | | **G4** | | | | | |
| --- | --- | --- | --- | --- | --- | --- | --- | --- | --- | --- | --- | --- | --- | --- | --- | --- | --- | --- | --- |
|  |  | **LI** | | **HI** | |  | **HI – LI** | **LI** | | **HI** | |  | **HI – LI** | **LI** | | **HI** | |  | **HI – LI** |
|  | ***N*** | ***M*** | *SD* | ***M*** | *SD* | *p* | Δ (%Δ) | ***M*** | *SD* | ***M*** | *SD* | p | Δ (%Δ) | ***M*** | *SD* | ***M*** | *SD* | *p* | Δ (%Δ) |
| Time [%] | 9 | **17.1** | 4.0 | **2.3** | 2.6 | <.01 | -14.8 pp | **36.0** | 10.0 | **50.7** | 8.9 | <.01 | 14.7 pp | **27.1** | 8.4 | **27.4** | 9.5 | .34 | 0.3 pp |
| CL [m] | 9 | **3.2** | 0.3 | **4.4** | 0.2 | .01 | 1.2 (38%) | **7.8** | 0.5 | **8.8** | **0.3** | <.01 | 1 (13%) | **8.8** | 0.3 | **10.3** | 0.5 | <.01 | 1.5 (17%) |
| CR [cpm] | 9 | **43.7** | 2.4 | **52.9** | 2.9 | .01 | 9.2 (21%) | **28.8** | 1.2 | **31.5** | **0.9** | <.01 | 2.7 (9%) | **36.0** | 1.6 | **37.8** | 1.2 | <.01 | 1.8 (5%) |
| CyT [s] | 9 | **1.39** | 0.07 | **1.14** | 0.07 | <.01 | -0.3 (-18%) | **2.09** | 0.08 | **1.91** | **0.05** | <.01 | -0.18 (-9%) | **1.68** | 0.07 | **1.59** | 0.05 | <.01 | 0.09 (-5%) |
| CT_ski_ [ms] | 9 | **800** | 55 | **617** | 57 | <.01 | -183 (-23%) | **1184** | 52 | **1062** | **46** | <.01 | -122(-10%) | **960** | 50 | **888** | 42 | <.01 | -72 (%) |
| CT_pole_ [ms] | 9 | **672** | 36 | **438** | 20 | <.01 | -234 (35%) | **410** | 33 | **356** | **24** | <.01 | -54 (-13%) | **402** | 38 | **354** | 39 | <.01 | -48(-12%) |
| %CT_ski_ [%] | 9 | **57.6** | 3.9 | **54.0** | 5.0 | .15 | -3.6 pp | **56.5** | 3.8 | **55.6** | **2.4** | .11 | -0.9 pp | **57.3** | 1.3 | **55.9** | 1.4 | <.01 | -1.4 pp |
| %CT_pole_ [%] | 9 | **48.4** | 2.6 | **38.4** | 1.7 | <.01 | -10 pp | **19.6** | 2.4 | **18.6** | **1.2** | .02 | -1 pp | **24.0** | 2.3 | **22.3** | 2.4 | <.01 | -1.7 pp |
| P_cycle_ [Watt] | 8 | **243** | 16 | **425** | 36 | <.01 | 182 (75%) | **213** | 16 | **332** | 32 | <.01 | 119 (56%) | **171** | 15 | **210** | 20 | <.01 | 39 (23%) |
| %P_pole_ [%] | 8 | **44.2** | 4.6 | **56.6** | 4.0 | .01 | 12.4 pp | **61.5** | 5.3 | **59.7** | 4.8 | .42 | -1.8 pp | **55.0** | 6.5 | **53.8** | 5.8 | .55 | -1.2 pp |
| P_pole_ [Watt] | 8 | **107** | 4.9 | **241** | 9.6 | <.01 | 134 (125%) | **131** | 6.9 | **198** | 9.5 | <.01 | 67 (51%) | **94** | 6.1 | **113** | 6.6 | <.01 | 19 (20%) |
| %P_ski_ [%] | 8 | **55.8** | 4.6 | **43.4** | 4.0 | .01 | -12.4 pp | **38.3** | 5.3 | **40.3** | 4.8 | .42 | 2 pp | **45.0** | 6.5 | **46.2** | 5.8 | .55 | 1.2 pp |
| P_ski_ [Watt] |  | **136** | 11 | **184** | 17 | <.01 | 48 (35%) | **82** | 11 | **134** | 16 | <.01 | 52 (63%) | **77** | 11 | **97** | 12 | <.01 | 20 (26%) |
| %P_poleleft_ [%] | 8 | **46.7** | 4.5 | **46.5** | 2.5 | .98 | -0.2 pp | **47.9** | 4.3 | **49.0** | 3.3 | .33 | 1.1 pp | **46.4** | 6.4 | **46.1** | 5.5 | .58 | -0.3 pp |
| %P_polerigth_ [%] | 8 | **53.3** | 4.5 | **53.5** | 2.5 | .98 | 0.2 pp | **52.1** | 4.3 | **51.0** | 3.3 | .33 | -1.1 pp | **53.6** | 6.4 | **53.9** | 5.5 | .58 | 0.3 pp |
| **Change: Lap 7 – Lap 1** |  |  |  |  |  |  |  |  |  |  |  |  |  |  |  |  |  |  |  |
| %P_pole_ [pp] | 8 | **-1.2** | 2.9 | **NA** | NA | *NA* | NA | **-8.4*** | 4.2 | **-8.3*** | 3.6 | .67 | 0.1 | **-6.9*** | 4.7 | **-7.4*** | 5.6 | .85 | -0.5 pp |
| %P_ski_ [pp] | 8 | **1.2** | 2.9 | **NA** | NA | *NA* | NA | **8.4*** | 4.2 | **8.3*** | 3.6 | .67 | -0.1 | **6.9*** | 4.7 | **7.4*** | 5.6 | .85 | 0.5 pp |
| %P_poleleft_ [pp] | 8 | **-0.8** | 2.1 | **NA** | NA | *NA* | NA | **0.6** | 1.3 | **0.2** | 1.5 | .56 | -0.4 | **-0.6** | 2.7 | **0.0** | 2.1 | .44 | 0.6 pp |
| %P_polerigth_ [pp] | 8 | **0.8** | 2.1 | **NA** | NA | *NA* | NA | **-0.6** | 1.3 | **-0.2** | 1.5 | .56 | 0.4 | **0.6** | 2.7 | **0.0** | 2.1 | .44 | -0.6 pp |
| *Note. N* = number of participants, cpm = cycles per minute, Δ = difference in mean values between HI and LI, %Δ = percentage of difference in mean values between HI and LI relative to LI, P_cycle_ = mean power for cycle, %P_pole_ = relative power from poling compared with P_cycle_, %P_ski_ = relative power from ski push-offs compared with P_cycle_, %P_poleleft_ = relative power from left pole compared with P_pole_, %P_polerigth_ = relative power from right pole compared with P_pole_, NA = not applicable due to lack of skiers using G2 during Lap 7. * = significant time-dependent change from Lap 7 to Lap 1. Non-significant numbers are highlighted in light grey. | | | | | | | | | | | | | | | | | | | |

**Supplementary Table 2** Selection of sub-techniques (in % of time), temporal patterns in form of cycle length (CL), cycle rate (CR), cycle time (CyT), contact time for poles (CT_ski_) and skis (CT_ski_), and power variables for the low-intensity (LI) versus high-intensity (HI) sessions, with means (*M*), standard deviations (*SD*), and *p* values for all laps (only Laps 1, 3, 5, and 7 for power distribution) for each segment. Only data for the sub-techniques mostly used in each segment are given, and data for skiers who spent less than 6 s on a sub-technique were disregarded.

| **Segment** | | **Segment 1 (5%)** | | | | | | **Segment 2 (2%)** | | | | | | **Segment 3 (12%)** | | | | | | | | | | | |
| --- | --- | --- | --- | --- | --- | --- | --- | --- | --- | --- | --- | --- | --- | --- | --- | --- | --- | --- | --- | --- | --- | --- | --- | --- | --- |
| **Subtechnique** | | **G3** | | | | | | **G4** | | | | | | **G2** | | | | | | **G3** | | | | | |
| **Intensity** | | **LI** | | **HI** | |  | **HI – LI** | **LI** | | **HI** | |  | **HI – LI** | **LI** | | **HI** | |  | **HI – LI** | **LI** | | **HI** | |  | **HI – LI** |
|  | ***N*** | ***M*** | *SD* | ***M*** | *SD* | *p* | Δ (%Δ) | ***M*** | *SD* | ***M*** | *SD* | *p* | Δ (%Δ) | ***M*** | *SD* | ***M*** | *SD* | *p* | Δ (%Δ) | ***M*** | *SD* | ***M*** | *SD* | *p* | Δ (%Δ) |
| Time [%] | 9 | **85.2** | 24.3 | **88.0** | 27.4 | .39 | 2.8 pp | **90.2** | 5.3 | **91.9** | 2.9 | .33 | 1.7 pp | **63.5** | *20.3* | **7.7** | 9.1 | *<.01* | -55.8 pp | **25.3** | *20.6* | **82.8** | 8.1 | <.01 | 57.5 pp |
| CL [m] | 9 | **8.2** | 0.3 | **10.0** | 0.5 | <.01 | 1.8 (22%) | **9.3** | 0.4 | **10.6** | 0.4 | <.01 | 1.3 (14%) | **3.1** | 0.2 | **4.4** | 0.3 | *<.01* | 1.3 (42%) | **5.4** | 0.8 | **7.1** | 0.2 | <.01 | 1.7 (32%) |
| CR [cpm] | 9 | **28.7** | 1.1 | **30.3** | 1.4 | <.01 | 1.6 (6%) | **35.9** | 1.7 | **37.8** | 1.3 | <.01 | 1.9 (5%) | **43.5** | 2.3 | **52.8** | 3.0 | *<.01* | 9.3 (21%) | **29.6** | 1.1 | **33.4** | 1.0 | <.01 | 3.8 (13%) |
| CyT [s] | 9 | **2.10** | 0.08 | **2.0** | 0.10 | <.01 | -0.1 (-5%) | **1.68** | 0.08 | **1.59** | 0.05 | .09 | 0.1 (-5%) | **1.39** | 0.07 | **1.14** | 0.07 | *<.01* | -0.25 (-18%) | **2.04** | 0.08 | **1.81** | 0.05 | <.01 | -0.23 (-11%) |
| CT_ski_ [ms] | 9 | **1200** | 51 | **1116** | 50 | <.01 | -84 (-7%) | **958** | 52 | **888** | 44 | <.01 | 70 (-7%) | **790** | 47 | **615** | 20 | *<.01* | -175 (-22%) | **982** | 71 | **980** | 45 | .93 | -2 (-0.2%) |
| CT_pole_ ms] | 9 | **396** | 31 | **341** | 24 | <.01 | -55 (-14%) | **391** | 29 | **350** | 38 | <.01 | -41 (-11%) | **677** | 34 | **438** | 20 | *<.01* | -239 (-35%) | **504** | 28 | **382** | 25 | <.01 | -122 (-24%) |
| %CT_ski_ [%] | 9 | **57.1** | 2.33 | **55.6** | 1.61 | <.01 | -1.5 pp | **57.1** | 1.36 | **55.8** | 1.28 | <.01 | -1.3 pp | **57.0** | 1.66 | **53.8** | 3.47 | .19 | -3.2 | **48.1** | 3.68 | **54.1** | 2.63 | <.01 | 6.0 |
| %CT_pole_ [%] | 9 | **18.8** | 1.02 | **17.0** | 0.87 | <.01 | -1.8 pp | **23.3** | 1.67 | **22.0** | 2.10 | <.01 | -1.3 pp | **48.8** | 1.51 | **38.4** | 1.50 | *<.01* | -10.4 | **24.7** | 0.98 | **21.1** | 1.17 | <.01 | -3.6 |
| P_cycle_ [Watt] | 8 | **209** | 14 | **269** | 18 | <.01 | 60 (29%) | **164** | 11 | **196** | 14 | <.01 | 32 (20%) | 245 | 17 | 425 | 36 | *<.01* | 180 (74%) | 243 | 17 | 428 | 30 | <.01 | 185 (76%) |
| %P_pole_[%] | 8 | **64.3** | 6.8 | **64.9** | 6.0 | .83 | 0.6 pp | **56.6** | 8.0 | **54.6** | 5.9 | .54 | -2.0 pp | **43.6** | 4.8 | **56.6** | 4.0 | .20 | 13 pp | **46.6** | 6.5 | **53.9** | 3.9 | .09 | 7.3pp |
| P_pole_ [Watt] | 8 | **134** | 14.2 | **175** | 16.1 | <.01 | 40.2 (30%) | **93** | 13.1 | **107** | 11.6 | .01 | 14 (15%) | **107** | 11.8 | **241** | 17.0 | *<.01* | 134 (125%) | **113** | 15.8 | **231** | 16.7 | <.01 | 118 (103%) |
| %P_ski_ [%] | 8 | **35.7** | 6.8 | **35.1** | 6.0 | .83 | -0.6 pp | **43.3** | 5.9 | **45.4** | 5.9 | .54 | 2.1 pp | ***56.4*** | *4.8* | ***43.3*** | *4.0* | .20 | -13.1 pp | **53.4** | 6.5 | **46.1** | 3.9 | .09 | -7.3pp |
| P_ski_ [Watt] | 8 | **75** | 14 | **94** | 16 | <.01 | 20 (27%) | **71** | 10 | **89** | 12 | <.01 | 18 (25%) | **138** | 12 | **184** | 17 | *<.01* | 46 (33%) | **130** | 16 | **197** | 17 | <.01 | 68 (52%) |
| %P_poleleft_ [%] | 8 | **47.9** | 4.3 | **49.0** | 3.7 | .27 | 1.1 pp | **46.0** | 6.8 | **46.0** | 6.0 | .90 | 0.0 pp | **46.8** | 3.9 | **46.6** | 2.4 | .15 | -0.2 pp | **48.0** | 4.0 | **49.1** | 2.9 | .35 | 1.1pp |
| %P_polerigth_ [%] | 8 | **52.1** | 4.3 | **51.0** | 3.7 | .27 | -1.1 pp | **53.9** | 6.8 | **54.0** | 6.0 | .90 | 0.1 pp | **53.2** | 3.8 | **54.4** | 2.4 | .15 | 1.2 pp | **52.0** | 4.0 | **50.9** | 2.9 | .35 | -1.1pp |
| *Note. N* = number of participants, cpm = cycles per minute, Δ = difference in mean value between HI and LI, %Δ = percentage of difference in mean value between HI and LI relative to LI, P_cycle_ = mean power for cycle, %P_pole_ = relative power from poling compared with P_cycle_, %P_ski_ = relative power from ski push-offs compared with P_cycle_, %P_poleleft_ = relative power from left pole compared with P_pole_, %P_polerigth_ = relative power from right pole compared with P_pole_. Non-significant numbers are highlighted in light grey. | | | | | | | | | | | | | | | | | | | | | | | | | |
